# Hypoglycaemia Prevention, Awareness of Symptoms, and Treatment (HypoPAST): protocol for a 24-week hybrid type 1 randomised controlled trial of a fully online psycho-educational programme for adults with type 1 diabetes

**DOI:** 10.1186/s13063-024-08556-1

**Published:** 2024-10-29

**Authors:** Jennifer A. Halliday, Elizabeth Holmes-Truscott, Sharmala Thuraisingam, Uffe Søholm, Mary Lou Chatterton, Sienna Russell-Green, Eric O, Sof Andrikopoulos, Taryn Black, Susan Davidson, Glen Noonan, Renza Scibilia, Virginia Hagger, Christel Hendrieckx, Cathrine Mihalopoulos, James A. M. Shaw, Vincent L. Versace, Sophia Zoungas, Timothy C. Skinner, Jane Speight, on behalf of the HypoPAST Research Group, Jennifer A. Halliday, Elizabeth Holmes-Truscott, Sharmala Thuraisingam, Uffe Søholm, Mary Lou Chatterton, Sienna Russell-Green, Eric O, Sof Andrikopoulos, Taryn Black, Susan Davidson, Glen Noonan, Renza Scibilia, Virginia Hagger, Christel Hendrieckx, Cathrine Mihalopoulos, James A. M. Shaw, Vincent L. Versace, Sophia Zoungas, Timothy C. Skinner, Shaira Baptista, Chatpakorn Prasertsung, Alison Robinson

**Affiliations:** 1 School of Psychology, Deakin University, Geelong, VIC Australia; 2 The Australian Centre for Behavioural Research in Diabetes, Diabetes Victoria, Melbourne, VIC Australia; 3 Institute for Health Transformation, Deakin University, Geelong, VIC Australia; 4 Australian Centre for Accelerating Diabetes Innovations, The University of Melbourne, Melbourne, VIC Australia; 5 Deakin Rural Health, Deakin University, Warrnambool, Australia; 6 School of Public Health and Preventive Medicine, Monash University, Melbourne, VIC Australia; 7 Digital Engagement, Digital Service, Deakin University, Geelong, VIC Australia; 8 Australian Diabetes Society, Sydney, NSW Australia; 9 Diabetes Australia, Turner, ACT Australia; 10 Australian Diabetes Educators Association, Turner, ACT Australia; 11 Diabetes Victoria, Carlton, VIC Australia; 12 Breakthrough T1D, New York, USA; 13 School of Nursing and Midwifery, Deakin University, Geelong, VIC Australia; 14 Translational and Clinical Research Institute, Newcastle University, Newcastle Upon Tyne, UK; 15 Institute of Psychology, Copenhagen University, Copenhagen, Denmark

**Keywords:** Hypoglycaemia, Fear of hypoglycaemia, Type 1 diabetes, Psycho-educational training

## Abstract

**Background:**

Management of type 1 diabetes (T1D) requires the use of insulin, which can cause hypoglycaemia (low blood glucose levels). While most hypoglycaemic episodes can be self-treated, all episodes can be sudden, inconvenient, challenging to prevent or manage, unpleasant and/or cause unwanted attention or embarrassment. Severe hypoglycaemic episodes, requiring assistance from others for recovery, are rare but potentially dangerous. Repeated exposure to hypoglycaemia can reduce classic warning symptoms (‘awareness’), thereby increasing risk of severe episodes. Thus, fear of hypoglycaemia is common among adults with T1D and can have a negative impact on how they manage their diabetes, as well as on daily functioning, well-being and quality of life. While advances in glycaemic technologies and group-based psycho-educational programmes can reduce fear, frequency and impact of hypoglycaemia, they are not universally or freely available, nor do they fully resolve problematic hypoglycaemia or associated worries. This study aims to determine the effectiveness of a fully online, self-directed, scalable, psycho-educational intervention for reducing fear of hypoglycaemia: the Hypoglycaemia Prevention, Awareness of Symptoms, and Treatment (HypoPAST) programme.

**Methods:**

A 24-week, two-arm, parallel-group, hybrid type 1 randomised controlled trial, conducted remotely (online and telephone). Australian adults (≥ 18 years) with self-reported T1D and fear of hypoglycaemia will be recruited, and allocated at random (1:1) to HypoPAST or control (usual care). The primary outcome is the between-group difference in fear of hypoglycaemia (assessed using HFS-II Worry score) at 24 weeks. A sample size of *N* = 196 is required to detect a 9-point difference, with 90% power and allowing for 30% attrition. Multiple secondary outcomes include self-reported psychological, behavioural, biomedical, health economic, and process evaluation data. Data will be collected at baseline, 12 and 24 weeks using online surveys, 2-week ecological momentary assessments, website analytics and semi-structured interviews.

**Discussion:**

This study will provide evidence regarding the effectiveness, cost-effectiveness and acceptability of a novel, online psycho-educational programme: HypoPAST. Due to the fully online format, HypoPAST is expected to provide an inexpensive, convenient, accessible and scalable solution for reducing fear of hypoglycaemia among adults with T1D.

**Trial registration:**

Australian and New Zealand Clinical Trials Registry (ANZCTR): ACTRN12623000894695 (21 August 2023).

**Supplementary Information:**

The online version contains supplementary material available at 10.1186/s13063-024-08556-1.

## Administrative information

**Table:** 

Title	Hypoglycaemia Prevention, Awareness of Symptoms, and Treatment (HypoPAST): Protocol for a 24-week hybrid type 1 randomised controlled trial of a fully online psycho-educational programme for adults with type 1 diabetes
Trial registration	Australian and New Zealand Clinical Trials Registry: ACTRN12623000894695 (Registered: 21 August 2023). All with all items from the WHO Trial Registration Data Set can be found in the Australian and New Zealand Clinical Trials Registry: https://www.anzctr.org.au/Trial/Registration/TrialReview.aspx?id=385968&isReview=true
Protocol version	Protocol version 1.1 [24 January 2024].
Funding	This project is funded by the Medical Research Future Fund (MRFF) Targeted Translation Research Accelerator (TTRA). In-kind support is provided by our partners: Australian Diabetes Educators Association, Australian Diabetes Society, Diabetes Australia, Diabetes Victoria, and uMotif, and academic partners: Deakin University, La Trobe University, Monash University, and Newcastle University. EH-T, CH and JSp are supported by the core funding to the Australian Centre for Behavioural Research in Diabetes (ACBRD) provided by the collaboration between Diabetes Victoria and Deakin University. VLV is supported by the Rural Health Multidisciplinary Training (RHMT) training programme (funded by the Australian Government Department of Health and Aged Care). SZ receives funding from NHMRC, MRFF, and the Victorian Department of Health and Human Services. We acknowledge peer-reviewed funding from Diabetes UK which enabled creation of the ‘my hypo compass’ educational programme which was provided by Newcastle University to facilitate generation of the novel HypoPAST programme.
Author details	Jennifer A Halliday^1,2,3^, Elizabeth Holmes-Truscott^1,2,3,4^, Sharmala Thuraisingam^2,5^, Uffe Søholm^1,2,3^, Mary Lou Chatterton^6^, Sienna Russell-Green^1,2^, Eric O^7^, Sof Andrikopoulos^8^, Taryn Black^9^, Susan Davidson^10^, Glen Noonan^11^, Renza Scibilia^12^, Virginia Hagger^3,13^, Christel Hendrieckx^1,2^, Cathrine Mihalopoulos^6^, James AM Shaw^14^, Vincent L Versace^4,5^, Sophia Zoungas^6^, Timothy C Skinner^1,2,15^, and Jane Speight^1,2,3^, on behalf of the HypoPAST Research Group 1School of Psychology, Deakin University, Geelong, Victoria, Australia 2The Australian Centre for Behavioural Research in Diabetes, Diabetes Victoria, Melbourne, Victoria, Australia 3Institute for Health Transformation, Deakin University, Geelong, Victoria, Australia 4 Australian Centre for Accelerating Diabetes Innovations, The University of Melbourne, Melbourne, VIC, Australia 5Deakin Rural Health, Deakin University, Warrnambool, Australia 6 School of Public Health and Preventive Medicine, Monash University, Melbourne, Victoria, Australia 7 Digital Engagement, Digital Service, Deakin University, Geelong, Victoria, Australia 8Australian Diabetes Society, Sydney, NSW, Australia 9Diabetes Australia, Turner, ACT, Australia 10 Australian Diabetes Educators Association, Turner, ACT, Australia 11 Diabetes Victoria, Carlton, Victoria, Australia 12Breakthrough T1D, New York, USA 13School of Nursing and Midwifery, Deakin University, Geelong, Victoria, Australia 14Translational and Clinical Research Institute, Newcastle University, Newcastle Upon Tyne, UK 15Institute of Psychology, Copenhagen University, Copenhagen, Denmark
Name and contact information for the trial sponsor	Not applicable, as the study does not involve an unapproved therapeutic good requiring a Clinical Trial Notification.
Role of sponsor and funding body	The funder, The Medical Research Future Fund (MRFF) Targeted Translation Research Accelerator, did not contribute to the development of this trial protocol, and will not be involved in the conduct of the trial data collection, analysis, interpretation or write-up of findings.

## Introduction

### Background and rationale

People with type 1 diabetes (T1D) need insulin for survival. However, even with modern insulins and delivery systems, exogenous insulin delivery cannot fully mimic endogenous insulin supply [1]. Hypoglycaemia (low blood glucose) remains a common side effect, caused by relative insulin excess in the absence of sufficient blood glucose. Around 20% of adults with T1D have experienced severe hypoglycaemia (requiring assistance from another person for recovery) in the past 6 months [2]. Hypoglycaemia can be unpleasant, sudden, embarrassing, unpredictable and dangerous (e.g. causing accident or injury) [2, 3]. If undetected and untreated, hypoglycaemia can be life-threatening and associated with adverse outcomes [4]. Repeated exposure to hypoglycaemia can lead to impaired awareness of hypoglycaemia symptoms, increasing a person’s risk of severe episodes, as they have reduced visibility of onset, reducing the window of opportunity for self-treatment [3]. Unsurprisingly, repeated exposure to hypoglycaemia, severe hypoglycaemia, and impaired awareness of hypoglycaemia symptoms can lead to distress and hypoglycaemia-related anxiety, known as ‘fear of hypoglycaemia’ [3, 5, 6].

Fear of hypoglycaemia can compromise diabetes self-management (e.g. maintaining higher glucose levels) and quality of life (e.g. limiting spontaneity, independence and activities in which hypoglycaemia may be a risk or problematic if it occurred) [7]. ‘Worries about low blood glucose’ are consistently among the top five problem areas experienced by adults with T1D, with 75% experiencing it as at least a ‘mild problem’ and 40% as a ‘moderate problem’ [8–11]. Fear of hypoglycaemia can be triggered by the anticipation, frequency, severity, and sequalae of hypoglycaemia, while asleep or awake, and by impaired awareness of hypoglycaemia [3]. Further, a US study found that 26% of adults with T1D met the diagnostic criteria for post-traumatic stress disorder in relation to their experience of hypoglycaemia [12]. Thus, managing fear of hypoglycaemia requires attention to multiple, modifiable risk factors for hypoglycaemia, in addition to general coping, and anxiety-reduction strategies.

The main therapeutic option for reducing fear of hypoglycaemia is the use of diabetes technologies, such as continuous glucose monitoring (CGM) and automated insulin delivery, which can reduce problematic hypoglycaemia [13, 14]. However, they do not necessarily eliminate (fear of) hypoglycaemia [15–17], and the psychological and cognitive burden can be increased by continual attachment to devices, visibility of data, and information overload [18, 19]. Further, financial burden remains a considerable barrier to widespread use [20, 21]. Several group-based psycho-educational programmes, involving glucose awareness training, have shown benefits for reducing fear of hypoglycaemia [3, 22–24]. However, none of these studies have measured fear of hypoglycaemia as a primary outcome and there remains a gap in the literature regarding the effectiveness of psycho-educational programmes in reducing fear of hypoglycaemia among people with high fear at baseline [25]. Furthermore, as such programmes are resource intensive, they are not implemented in routine clinical care in Australia or most other countries. Despite Australia’s National Health and Medical Research Council (NHMRC) T1D guidelines asserting the urgent need for minimal resource interventions to prevent severe hypoglycaemia [26], no Australian studies have focused on this unmet need. Furthermore, numerous international studies now demonstrate that adults with T1D need and want more clinical, psychological and educational support for hypoglycaemia [27–29].

### Objectives

This study aims to examine the effectiveness of a fully online, self-directed, psycho-educational programme for reducing fear of hypoglycaemia among adults with T1D: HypoPAST, which stands for ‘*Hypo*glycaemia *P*revention, *A*wareness of *S*ymptoms, and *T*reatment’. There are four objectives:

1. To assess the effect of HypoPAST on fear of hypoglycaemia among adults with T1D;
2. To assess the effect of HypoPAST on secondary psychological, clinical and behavioural outcomes;
3. To assess the cost-effectiveness of HypoPAST; and
4. To explore the reach, acceptability, usability and sustainability of HypoPAST to adults with T1D, and understand the extent to which learnings from the programme are implementable in the ‘real-world’, using a mixed-methods process evaluation.

### Trial design

This study is a two-arm, parallel-group, hybrid type 1 mixed-method randomised controlled trial (RCT) [30]. Eligible participants will be allocated on a 1:1 basis to intervention (HypoPAST) or control (usual care). The trial is designed to determine the superiority of HypoPAST compared to usual care.

Data will be collected at baseline, mid-trial (week 12) and end-trial (week 24): survey data collected at baseline, mid-trial and end-trial; ecological momentary assessment (EMA) data collected twice daily for 2 weeks at baseline and end-trial; interview data collected at end-trial; and website analytics collected during implementation (weeks 0–23).

## Methods

### Study setting

The study will be conducted in Australia, using primarily online methods. Participants will use their own device(s) (i.e. laptop, desktop, smartphone, and/or tablet computer) from a physical location of their choice.

Platform O, a Deakin University owned and developed e-research platform, will be used to host the online intervention and automate data collection and email reminder systems. Platform O is a widely used evidence-based research tool which provides an end-to-end e-Research solution and complies with Australian ethics requirements [31, 32]. It includes a dynamic intervention builder, which allows researchers to develop highly interactive contents without programming knowledge, including integration with Qualtrics (Provo, UT), Microsoft PowerPoint and Word, PDFs, Videos (You Tube and Vimeo), third party games and animations and text and images. The platform can be used on any device; however, HypoPAST participants will be encouraged to use a tablet, laptop or desktop computer to access the HypoPAST intervention, due to their larger display sizes.

Survey data will be collected via Qualtrics, web analytics will be collected via Google Analytics and AUDCI framework and telephone interviews will be conducted via Microsoft Teams. EMA data will be collected via an ‘app’ using a smartphone or tablet device. The app was developed for a study of hypoglycaemia-related experiences (Hypoglycemia MEasurement, ThResholds and ImpaCtS:HypoMETRICS) among adults with T1D and insulin-treated type 2 diabetes [33], and adapted for HypoPAST. The app is administered via the uMotif Limited platform (umotif.com), which is used widely for EMA in person-centred data capture studies.

### Eligibility criteria

Eligible participants will be adults (≥ 18 years) living in Australia; with self-reported T1D; with self-reported fear of hypoglycaemia (response to a single item from the Problem Areas in Diabetes indicating that worry about low blood glucose is at least a ‘moderate problem’) scale [11, 34]; and access to the internet via one of the following combinations: (1) a smartphone and tablet, (2) tablet only or (3) desktop or laptop and tablet or smartphone. Members of the HypoPAST Type 1 Diabetes Lived Experience Steering Group (comprising adults with T1D) will be excluded from participation in the trial.

### Informed consent

Potential participants will be directed to visit the recruitment website (acbrd.org.au/take-part/hypopast-rct), which will include brief descriptions of the HypoPAST study and intervention, the plain language statement and a hyperlink to a Qualtrics survey for participant consent and eligibility screening enabling self-enrolment into the study.

### Interventions

#### Intervention description

HypoPAST is an online psycho-educational training programme informed by several group-based programmes [23, 24, 35–37]; behavioural, psychological, educational and clinical insights from a multidisciplinary team; and lived experience insights from a Steering Group of adults with T1D. Participants allocated to the intervention group will receive email instructions about how to access the HypoPAST programme via the website (hypopast.org.au). They will be advised that HypoPAST is intended to enhance, not replace, the individualised medical advice of their diabetes health professional(s); and to continue with their usual diabetes management, clinical care and support during the trial. Participants will be advised that the recommendation is to complete all 7 modules and to do so over 4–8 weeks. This recommendation is made to provide participants with opportunity to digest the content and practice skills. In total, the programme is estimated to take 4 to 8 h (about 30 to 60 min per module), depending on the number of modules undertaken and the time an individual spends on activities and practising skills between modules. As HypoPAST is self-guided, participants will choose how many and which modules they complete, in what order, how many times and the timeframe (e.g. in one or multiple sessions, in 1 day or over multiple days/weeks). There is no limit on how often they access HypoPAST and they will have access to the programme for the full 24 weeks of the trial.

**Fig. 1:**
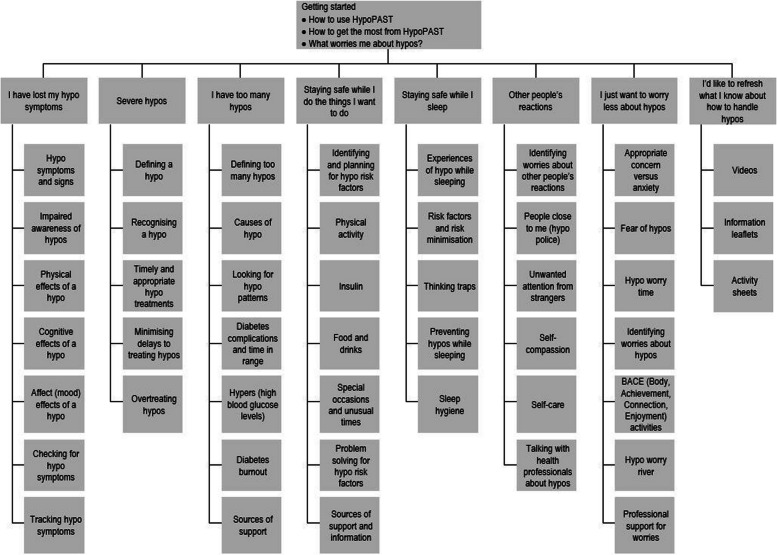
HypoPAST overview

Upon entry to the programme, the brief ‘Getting started’ module demonstrates how to make the most of the programme and enables participants, via self-assessment, to prioritise the modules most relevant to their needs. The HypoPAST programme comprises seven modules, covering topics such as timely and appropriate treatment of hypoglycaemia; personal risks for hypoglycaemia; recognising warning signs of hypoglycaemia; drivers of frequent hypoglycaemia; talking about hypoglycaemia with family, friends, health professionals and colleagues; hypoglycaemia while asleep; and managing worries. HypoPAST includes reflection and problem-solving activities enabling participants to discover for themselves how to strengthen their skills and develop preventative habits enabling them to reduce both their fear and their risk of hypoglycaemia (Fig. 1 ). In a separate section of the online platform, ‘My Resources’, participants will have access to information leaflets, activity sheets and videos related to topics covered in the modules.

#### Control description

There is currently no specific pathway for managing fear of hypoglycaemia among adults with T1D in Australia. Thus, participants allocated to the control group will be advised to continue with their usual T1D management, clinical care and support, which is assumed to involve strategies for minimising problematic hypoglycaemia. This may include contact with their diabetes specialist or healthcare team and/or the National Diabetes Services Scheme (NDSS) Helpline. At trial end, they will be granted access to the HypoPAST intervention for 24 weeks.

#### Strategies to improve adherence to interventions

Intervention group participants will receive three automated email reminders to access the intervention, at 2, 11 and 23 weeks post-randomisation. Intervention uptake will be monitored during the trial.

#### Concomitant care

Concomitant care will not be prohibited during the trial. All participants will be advised to continue with their routine diabetes self-management, as discussed with their diabetes health professional. With permission of the participants, we will inform their primary diabetes health professional that they are participating in the study. Participants will be advised, in the plain language study information, to contact their diabetes health professional if they experience severe hypoglycaemia or another diabetes-related issue during the study.

#### Provisions for post-trial care

This study will not include administration, manipulation or investigation of the effects, of any pharmacological or therapeutic goods. Therefore, provisions for ancillary or post-trial care are not deemed necessary. However, adverse event monitoring will take place, per Australian Government requirements (see ‘Oversight and monitoring’). The study is covered by Deakin University’s clinical trial insurance. Participants will be notified, in the plain language information, that they will not receive payment/reimbursement for medical expenses if they experience a severe hypoglycaemic event or another health-related issue during the study.

### Outcomes

Table 1 details all outcomes, self-reported demographic and clinical characteristics to be collected, the method by which they will be collected, and the timepoint(s) at which they will be collected.

**Table 1:** Schedule of HypoPAST assessments

**Assessment**	**Measure (number of items)**	**Timepoint (week)**		
		**0**	**12**	**24**
***Eligibility criteria***				
Age (≥18 years); Self-reported diagnosis (type 1 diabetes); Country (Australia); ‘Worrying about low glucose reactions’ is at least a moderate problem (PAID item score >2); Internet access via one of the following combinations: (1) a smartphone and tablet, (2) tablet only or (3) desktop or laptop and tablet or smartphone; the HypoPAST Type 1 Diabetes Lived Experience Steering Group membership	Study-specific (5 items) plus 1 item from the Problem Areas in Diabetes (PAID) scale [11, 34]	X	-	-
***Stratification criteria***				
Gender: men vs women	Study-specific (1 item)	X	-	-
Primary glucose monitoring method: finger-prick vs CGM/isCGM	Study-specific (1 item)	X	-	-
***Demographic and clinical characteristics***				
Age at T1D diagnosis, diabetes complications, diabetes education, country of birth, Aboriginal Torres Strait Islander, language(s) at home, State/Territory, postcode, education, employment, relationship status, living with someone who can assist with a hypoglycaemic event	Study-specific (12 items)	X	-	-
Insulin use: administration method	Study-specific (1 item)	X	X	X
Glucose monitoring: recording and use of glucose checks	Study-specific (2 items)	X	X	X
History of severe hypoglycaemia	Study-specific (2 items)	X	X	X
Time of main sleep (e.g. night or day)	Study-specific (1 item)	X	-	X
***Primary outcome***				
Fear of hypoglycaemia	HFS-II Worry subscale (18 items) [61]	X	X	X
***Secondary outcomes: clinical, psychological and behavioural***				
Most recent HbA1c; target glucose range, comfortable glucose range, time above/below target range; frequency of glucose checks; number of daily doses/boluses; daily insulin units	Study-specific (13 items)	X	X	X
Awareness of hypoglycaemia symptoms	Gold score (1 item) [62]	X	X	X
	Technological awareness: study specific (1 item)	X	X	X
	Hypoglycaemia Awareness Questionnaire (HypoA-Q) Impaired Awareness subscale (Items 7-8, 10-12) (5 items) [63]	X	X	X
Hypoglycaemia symptom burden and response	Study-specific (2 items)	X	X	X
Hypoglycaemia frequency & severity: Overall (past week & past 6 months); While awake and while asleep (past 6 months)	HypoA-Q: Items 1-4, 15 and 16) (6 items) [63]	X	-	X
Behaviours associated with fear of hypoglycaemia	Hypoglycaemia Fear Survey II Short Form (HFS-SF): Avoidance & Maintain High subscales (5 items) [38]	X	X	X
Confidence in managing hypoglycaemia	Hypoglycaemia Confidence Scale (9 items) [64]	X	X	X
Hypoglycaemia-specific quality of life	Hypoglycaemia Impact Profile (HIP-12; 12 items) [56]	X	X	X
Attitudes to awareness of hypoglycaemia	Attitudes to Awareness of Hypoglycaemia scale (A2A; Items 4-19 only; 16 items) [65]	X	X	X
Perceptions and experiences of hypoglycaemia	Hypoglycaemia Cues Questionnaire (Hypo C-Q; Items 2-40 only; 39 items) [24]	X	X	X
Hypoglycaemia-specific post-traumatic stress	Primary Care PTSD Screen for DSM-5 adapted for hypoglycaemia (PC-PTSD-5a; 5 items) [66]	X	X	X
Diabetes distress	PAID scale (PAID-11; 11 items) [11]	X	X	X
Anxiety and depressive symptoms	Patient Health Questionnaire (PHQ-4; 4 items) [67]	X	X	X
**Ecological momentary assessments**				
Hypoglycaemia episodes and symptoms, fear of hypoglycaemia, fear of hyperglycaemia, sleep quality, mood, anxiety, productivity	HypoMETRICS ‘check-ins’ (24 items) and ‘flower motif’ (10 items) [42, 68]	X	-	X
***Cost-effectiveness***				
Health-related quality of life	Assessment of Quality of Life (AQOL-4D; 12 items) [41]	X	X	X
Health service and resource use, productivity	Study-specific (15 items)	X	X	X
***Process evaluation***				
Acceptability, usability and sustainability	Study-specific questions (39 items)	-	X	-
Therapeutic alliance	Mobile Agnew Relationship Measure (mARM; 25 items) [46]	-	X	-
Reach and fidelity	Website analytics e.g. login patterns; devices used to access the programme; frequency of accessing and time spent (minutes) on programme and its components.	Throughout 24-week intervention access period		
Acceptability, usability and sustainability	Semi-structured interview schedule: perceptions of HypoPAST (including usability and sustainability), experiences of making behavioural changes in ‘real life’ attributable to *HypoPAST*	-	-	X

#### Primary outcome

The primary outcome is the between-group difference at 24 weeks in fear of hypoglycaemia, assessed using the 18-item Worry subscale of the Hypoglycaemia Fear Survey, version 2 (HFS-II) [38]. We hypothesise a statistically significant difference of at least 9 points will be observed in the Worry score, favouring the intervention.

#### Secondary outcomes

##### Clinical, psychological and behavioural

At each timepoint, several clinical, psychological and behavioural secondary outcomes are assessed using validated and study-specific survey measures. These include IAH, hypoglycaemia frequency and severity, attitudes to hypoglycaemia awareness, avoidant behaviours related to fear of hypoglycaemia, hypoglycaemia-specific quality of life, hypoglycaemia related post-traumatic stress, diabetes distress, generalised anxiety symptoms and depressive symptoms. Further details of the methods for assessment are provided in Additional File 1. We hypothesise that clinically significant between-group differences will be observed by 24 weeks in these variables, favouring the intervention.

##### Health economics

At each timepoint, participants will complete a resource use questionnaire in the online survey, to collect information about the use of other healthcare resources used and lost productivity [39, 40]. They will also complete a generic health-related quality of life questionnaire, the Assessment of Quality of Life 4 dimension (AQoL-4D), to assess utility values and calculate quality adjusted life years (QALYs) [41]. We hypothesise that HypoPAST will be cost-effective from health sector and societal perspectives compared to the control (usual care), with an incremental cost-effectiveness ratio below the commonly used willingness to pay threshold of $50,000/quality-adjusted life year.

##### Ecological momentary assessments

Ecological momentary assessment (EMA) provides opportunities to collect data in ‘real time’ on experiences of hypoglycaemia (e.g. timing, glucose level, symptoms) and their impacts (e.g. on sleep quality, productivity, mood). The EMA comprises two parts, both collected via an app. The questions were previously trialled in the 10-week HypoMETRICS study of hypoglycaemia-related experiences among adults with T1D and insulin-treated type 2 diabetes [33, 42, 43].

For the EMA analysis, we hypothesise that significant between-group differences at end-trial will be observed in effects of person-reported hypoglycaemia on daily functioning scores, number of hypoglycaemic episodes, awareness of symptoms and hypoglycaemia burden, all favouring intervention.

##### Reach, acceptability, usability, fidelity and sustainability

A mixed-methods approach will be used to examine the reach of HypoPAST, its acceptability and usability to adults with T1D, their experiences of integrating learnings from HypoPAST into their lives, and their views about sustainability and roll-out of HypoPAST. A mixed-methods approach enables collection of both quantitative data (e.g. intervention uptake, survey responses) and qualitative data (e.g. semi-structured interviews). This process evaluation will draw upon two highly relevant and complementary frameworks: Behaviour Interventions using Technology [44] and Reach, Effectiveness, Adoption, Implementation, and Maintenance [45].

Measures of reach will include study registration rates; proportion of registrants meeting the eligibility criteria; method of referral into the study; demographic and clinical characteristics; and study attrition. Additionally, various website analytics will be collected (Table 1) to assess protocol fulfilment with the intervention (i.e. the proportion of participants who accessed the HypoPAST at least once). We will also examine any association(s) between participant outcome and type/duration of content accessed.

Intervention group participants will complete study-specific survey items about the intervention’s acceptability and usability (e.g. reasons for accessing/not accessing modules, ease of understanding, trustworthiness, convenience, presentation, likes and dislikes, suggestions for improvement, technical problems) and sustainability (e.g. recommendations to others, suggestions for referral into the programme, potential costs for access). They will also complete the mARM to measure therapeutic alliance with the HypoPAST programme [46].

A semi-structured interview schedule (Additional File 2) will build upon topics from the survey, with the intention to gain in-depth insights into (1) the intervention group participant’s views about the HypoPAST intervention, (2) their experiences implementing principles and strategies from the intervention into their daily lives and (3) their views about sustainability and roll-out of HypoPAST.

### Participant timeline

**Fig. 2:**
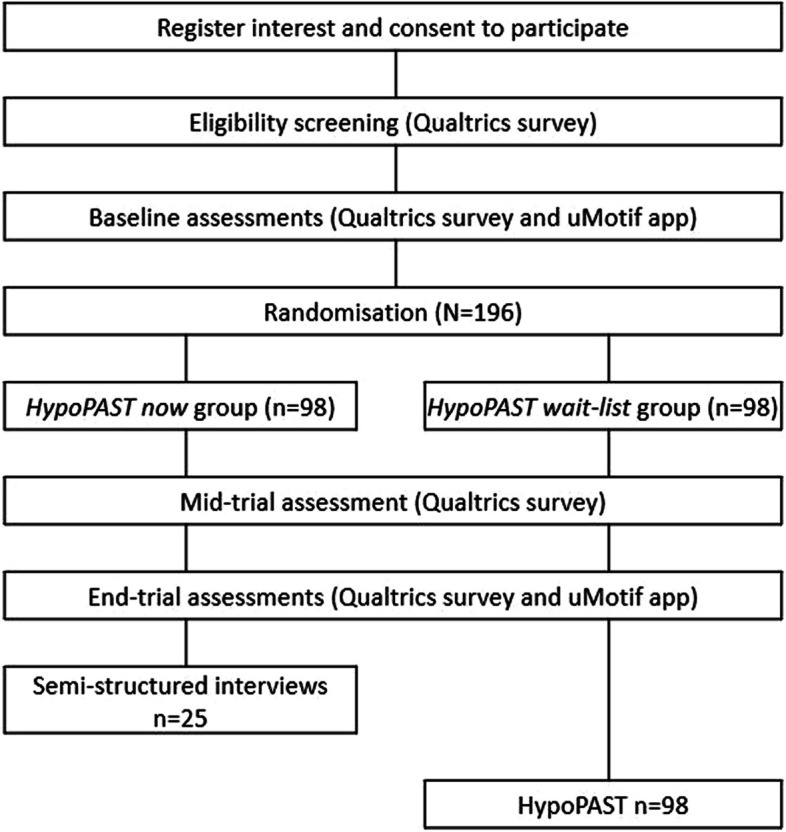
Flow diagram for HypoPAST randomised controlled trial

Self-enrolled, eligible participants will participate in 2 weeks of baseline data collection (survey plus 14-day EMA) before randomisation. Those who do not complete the baseline EMA (defined as missing ≥ 7 the 28 check-ins) will not proceed to randomisation. Those allocated to the intervention will be encouraged to complete the online HypoPAST training in the first 4 to 8 weeks, but will retain access throughout the full 24-week trial. Further data collections will take place at mid-trial (survey) and end-trial (EMA, survey, interviews). From entry to exit, participation will take ≤ 32 weeks, including all data collection. Figure 2 shows the study flow, and Table 2 shows the timeline of activities (e.g. enrolment, randomisation, intervention and data collection).

**Table 2:** Timeline for HypoPAST randomised controlled trial

	**Baseline: pre-randomisation**		**Implementation**				**End-trial**		
**Week:**	-2	-1	0	1 to 11	12	13 to 23	24	25	26+
**Screening, registration, randomisation**									
Consent and registration, eligibility screening survey	X								
Randomisation			X						
**Implementation**									
Intervention or usual care			X						
Intervention access expires (intervention group)							X		
**Data collection**									
Surveys^a^	X				X		X		
EMA daily check-ins (twice per day for 2 weeks)^b^	X						X		
Website analytics			X						
Semi-structured interview^c^									X

^a^Participants are encouraged to complete the online survey immediately upon receiving the hyperlink, however the surveys will remain open for three weeks to allow for late completion

^b^Baseline EMA will commence after the baseline survey is returned

^c^Approximate timing. Interviews will be scheduled after the participant returns their end-trial survey/EMA data, or after the window for survey/EMA data return closes (whichever occurs first)

### Sample size

The RCT sample size and statistical power calculations were conducted using STATA MP (StataCorp) ‘power twomeans’ sample size estimation package. Based on previous research [24], a standard deviation of 17 was used to estimate a sample size of *N* = 196 for this study. Given there is no minimal clinically important difference in fear of hypoglycaemia reported in the literature, the anticipated effect size was estimated from previous research [24]. To detect a between-group difference of nine points (standardised effect size of 0.5) in the primary outcome at 6 months, a total of 150 participants (75 per arm) is required. This sample size is a conservative estimate based on 1:1 group allocation for a two-sided test, at the 5% significance level, and a high level of power (90%). Allowing for 30% attrition at 24-week follow-up, the final required sample size is *N* = 196 participants (*N* = 150 × 1.3; *n* = 98 per study group).

For the qualitative sub-study, a purposive sample size of *n* = 25 was estimated to maximise diversity based on baseline survey responses (e.g. gender, age, education, glucose monitoring method, awareness of hypoglycaemia symptoms, and experience of severe hypoglycaemia).

### Recruitment

The primary recruitment method will be via the NDSS, an initiative of the Australian government administered by Diabetes Australia. Approximately 137,000 Australian adults with T1D are registered with the NDSS. NDSS staff will email the study invitation to a random of sample of adult registrants with T1D who have consented to being contacted about research (30%). Additional NDSS emails will be sent, if needed, to ensure timely achievement of the required sample size.

In addition, the study will be advertised via the e-newsletter, social media and website of the Australian Centre for Behavioural Research in Diabetes, and via direct email to adults with T1D who have previously requested to be notified of relevant research opportunities.

Participant demographics will be monitored throughout recruitment to examine diversity in representation. If needed, the study may be promoted in a more targeted manner (e.g. to encourage certain demographics to participate, within the channels listed above).

For the qualitative sub-study, participants will be recruited from intervention group participants using purposive sampling (see above). Engagement with the intervention will also be considered, as the interview questions require participants to comment on their experiences using and implementing the intervention into their daily lives.

### Randomisation

Participants will be allocated at random (1:1) to the intervention or control (usual care) arm. The allocation sequence will be generated by computer (via Qualtrics/Platform O) using randomly permuted block sizes. As stratification prevents imbalance in treatment groups for important variables thought to influence the outcome, randomisation will be stratified by:

1. Gender: men versus women. We anticipate that gender may influence the expression of emotional well-being (including the primary outcome and several secondary endpoints). If participants have a ‘non-binary’ or ‘another term’ gender identity, they will be allocated at random to either the male or female gender strata, so there is approximately equal representation of these smaller sub-groups in each arm.
2. Glucose monitoring method: finger-prick versus CGM or intermittent-scanned CGM (isCGM, also known as ‘flash’ monitoring). We anticipate that monitoring method may be associated with fear of hypoglycaemia, frequency and severity of hypoglycaemia, IAH and/or the extent to which participants benefit from HypoPAST (given their access or not to real-time glucose data).

The allocation sequence will be stored in the Deakin data centre server system with password protection, accessible only to the system architect of Platform O. Post-randomisation group allocations will be stored in the Deakin data centre and accessed from the backend of Platform O with a secure login, accessible only to the system architect and a research assistant who will be responsible for maintaining a password-protected codebook in Microsoft Excel.

Two weeks after self-enrolment in the study, participants will be notified of their group allocation by email, with instructions relevant to their allocation.

### Blinding

During data collection, all members of the HypoPAST research group will be blinded, with the exception of (1) a research assistant who will maintain a password-protected codebook for the purpose of data linkage between data sources (e.g. the app, Qualtrics surveys, website analytics) and be responsible for direct contact with participants (e.g. monitoring enquiries via the HypoPAST email, inviting people to participate in adverse event/process evaluation interviews, administering e-vouchers, informing the participants’ nominated diabetes health professionals about their participation, notifying participants of their randomised group allocations); and (2) the website architect responsible for Platform O (EO). Neither will conduct data analysis.

During data analysis, those analysing data for research questions 1 and 2 (ST, US, and VLV) and the investigators will remain blinded. For research question 3, the health economists (CM and MLC) will use blinded data to undertake their preliminary analyses of utility values and QALYs from the AQoL-4D, the self-reported health care resource use and lost productivity. However, the final economic evaluation will be conducted using unblinded data, as they will need to assign intervention costs to the appropriate group.

Due to the nature of the intervention and control conditions, participants will be aware of their group allocation.

Unblinding of the Project Manager is permissible if they are investigating a potential adverse event (see ‘Adverse event reporting’) via email or telephone interview. This may occur if an individual participant discloses information that identifies their group allocation. In such instances only the Project Manager will be unblinded. Breaches to blinding will be recorded and reported with the study findings.

### Data collection and management

#### Plans for assessment and collection of outcomes

Data will be collected in four ways:

- An online survey (administered via Qualtrics), to collect self-reported data on demographic and clinical characteristics; primary and secondary clinical, psychological and behavioural outcomes; and health economic outcomes. Intervention group participants will also answer questions about intervention acceptability and therapeutic alliance (mid-trial survey). The survey questions comprise a combination of validated scales and study-specific items, most of which are fixed-choice and some requiring text input (e.g. writing their age, most recent HbA1c, the number of hypos they have had in the past week, or specifying information where they have selected the ‘other’ response option) (Table 1 and Additional File 1). At mid-trial, intervention group participants are asked two qualitative questions related to intervention acceptability (suggestions for improvement and what they would tell others about HypoPAST). At each timepoint (0, 12 and 24 weeks), the survey is anticipated to take 45 min.
- An ‘app’, for EMA (administered via the uMotif platform) which collects self-reported data on clinical, psychological, behavioural and health economic outcomes (Table 1 and Additional File 1). Every day for 14 days (at two timepoints: pre-randomisation and end trial), participants will log their hypoglycaemia episodes and symptoms using the ‘motif flower’ in the app. They will complete one motif entry for each episode of hypoglycaemia and can complete the entry at any time on any day during the 2-week data collection period. Participants will also complete daily functioning ‘check-in’ survey questions twice daily (morning: between 06:00 to 12:00, and evening: 18:00 to 24:00) via the app. Each check-in will take about 5 min.
- Website analytics will be collected via Google Analytics and AUDCI framework, capturing user engagement with the HypoPAST intervention.
- Semi-structured, audio-recorded telephone interviews (approximately 30–45 min) at end-study, conducted by trained researchers with a sub-set of intervention group participants. The interviews will explore participant’s experiences using the intervention and implementing it into their daily lives, and their views about rollout of the intervention to other adults with diabetes. The interview will take place after the window for end-trial survey completion has closed (weeks 24 to 26), or the end-trial survey is returned, whichever comes first.

#### Plans to promote participant retention and complete follow-up

Participants will be offered a token of appreciation, recognising the commitment that it takes to participate in a 6-month trial. E-vouchers (AU$50) will be issued via email to participants after they complete the (1) mid-trial survey, (2) end-trial survey and EMA and (3) interview. This means, for example, an intervention group participant who takes part in both follow-ups and an interview will receive three e-vouchers (total value AU$150). Reminders will be scheduled to encourage engagement with the intervention and follow-up data return. Specifically, two email reminders (1 and 2 weeks after the initial invitation) will be automated to remind participants to complete the mid- and end-trial surveys. The app will send a daily push notification reminding participants to ‘check-in’, if their phone settings allow this.

Participants will be able to withdraw from the trial, for any reason, by emailing the study team prior to completion of the 24-week follow-up survey and the closing of the dataset for the primary analysis. Data collected until that timepoint will be retained in the analysis unless otherwise requested by the participant. Due to the online nature of the trial and due to feedback from DUHREC, the study team will not proactively enquire about participant’s reasons for withdrawal or discontinued engagement with the trial (e.g. non-return of follow-up data, refusal to participate in interview); however, reasons will be recorded if they are disclosed by the participant (e.g. via email or telephone contact).

#### Data management

As per the HypoPAST data management plan, during the trial, data will be collected by and held in the following locations: Qualtrics (survey responses), uMotif (app responses), Platform O (participant contact details and web analytics), Microsoft Teams (audio recordings of interviews), a transcribing company (audio recordings and transcripts of interviews). Once each phase of data collection is complete (i.e. baseline, 12-week follow-up, 24-week follow-up), or in the case of interviews, after each interview, the database will be locked, data de-identified and downloaded (e.g. from Qualtrics, uMotif, Platform O, Microsoft Teams) to the Deakin University secure network. Subsequently, any copies of the data held elsewhere will be destroyed (i.e. electronic files deleted).

Syncplicity (i.e. Sync and Share create via Deakin Research Data Store) will be used to share de-identified data with the researchers with primary responsibility for data analysis. Data will be retained on the Deakin University secure network for at least 15 years after the study findings are published, as required by law.

After the study findings are published, metadata will be placed in an open-access data repository (see ‘Availability of data and materials’).

#### Confidentiality

Unique participant identification codes will be assigned to participants. Personal identifiers will be removed from the data. A password-protected codebook will be maintained by a research assistant.

Individual participants will not be identified in reports of study findings, and data that could potentially be used to identify participants will not be included in the open-access meta-data or shared datasets.

### Statistical methods

#### Primary and secondary outcomes

##### Clinical, psychological and behavioural outcomes

To examine the effect of the intervention on primary and secondary outcomes (collected via survey), we will use an intention to treat approach where all randomised participants will be analysed according to their study group assignment (regardless of the extent to which intervention group participants engage with the intervention). Statistical analyses will be performed using STATA MP version 17 (StataCorp). Linear mixed models using restricted maximum likelihood estimation will be used to estimate group differences in the primary (HFS-II Worry scale score) and secondary clinical, psychological and behavioural outcomes (Table 1). As the primary and secondary outcomes will be measured longitudinally, the outcome at 12 and 24 weeks will be included in the model as the dependent variable. The outcome at baseline, time and an interaction between time and trial arm will be included as fixed effects in the models. Repeated outcome measures will be treated as random effects in the model and an unstructured variance–covariance structure assumed. Models will be adjusted by stratification factors: gender and glucose monitoring method. Transformations for skewed outcome measures will be considered. The estimated mean HFS-II Worry scale score at baseline, 12 and 24 weeks will be plotted for each trial arm with 95% confidence intervals. The estimated difference in mean HFS-II Worry score between arms at 12 and 24 weeks and associated 95% confidence intervals will be presented.

A similar modelling approach will be used to estimate group differences in hypoglycaemic events; however, a negative binomial mixed model will be used to account for repeated count data. In secondary analyses, the primary outcome will be adjusted by age, diabetes duration, HbA1c, severe hypoglycaemia episodes in the past 6 months, Gold score and insulin administration modality. Secondary outcomes will also be adjusted by these potential confounders where relevant. A per protocol analysis for the primary outcome will be conducted to estimate the treatment effect in those who engage with the intervention (defined as using 2 or more modules). A sensitivity analysis using multiple imputation may be conducted should there be between 10 and 40% missing data in the primary outcome and auxiliary variables available in the data set to explain the missingness. A second sensitivity analysis will be performed on the primary analysis to test the robustness of the ‘data missing at random’ assumption of mixed-linear models using pattern-mixture modelling.

##### Health economic outcomes

A cost-utility analysis will be undertaken from health sector and societal perspectives. The health sector perspective includes costs borne by the government as a third-party payer in addition to out-of-pocket costs incurred by patients when accessing healthcare. Detailed costing of the HypoPAST intervention will be performed using micro-costing methods. The number and types of additional health services used by participants over the period of the trial will be collected with a resource use questionnaire, and standard Australian unit costs will be applied. Total health sector costs will be calculated as the sum of intervention delivery and additional healthcare service use costs. The societal perspective adds the cost of lost productivity (absenteeism and presenteeism) to the health sector costs. Lost productivity will be measured with questions in the resource use questionnaire and valued with the human capital approach using an average Australian wage rate plus on-costs. The AQoL-4D utility values for each participant at each timepoint will be used to calculate QALYs [47] using the area under the curve method. The within-trial economic evaluation will measure and value any change in healthcare resource use and lost productivity and then compare any additional costs to additional QALYs through an incremental cost-effectiveness ratio. Bootstrapping will be used to determine confidence intervals for the incremental cost-effectiveness ratio and construct an acceptability curve to determine the cost-effectiveness of the intervention against the commonly used willingness to pay threshold of AU$50,000/QALY. Missing data will be explored and managed for the resource use and AQoL-4D questionnaires based on recommendations for analysis of trial-based economic evaluations with missing data [48]. Sensitivity analyses will be undertaken to evaluate the robustness of results with changes to costing or analytical assumptions. Scale-up and implementation costs as well as longer-term cost-effectiveness will be estimated based on population-wide modelling techniques using published epidemiological data. STATA (StataCorp) will be used for these analyses.

##### Ecological momentary assessment outcomes

To examine the effect of the intervention on EMA outcomes (daily functioning, number of hypoglycaemic episodes, awareness of symptoms and hypoglycaemia burden collected via an app), we will use linear mixed models (for continuous outcomes) and mixed negative binomial models (for count outcomes). Trial arm, time of day (morning/night) and timepoint (baseline or 24 weeks) will be included as fixed effects in the models. Participants will be included as random effects in the models and an unstructured variance–covariance structure used to account for repeated measures and the correlation in outcome within individuals. A separate mixed linear model will be used to explore whether the effect of person-reported hypoglycaemia on daily functioning domains (fear of hypoglycaemia, x, y) differs between trial arms. The independent variable will be person-reported hypoglycaemia, and an interaction between this variable and trial arm (intervention or control) will be included in the model. If sufficient data, we will also assess additional models exploring effects of person-reported hypoglycaemia subtypes (i.e. how the episodes where detected and managed) and whether number of reported hypoglycaemia episodes, awareness (in terms of symptoms) and hypoglycaemia burden differ between trial arms. Models will be adjusted as for the primary outcome. Similarly, missing data will be managed as for the primary objective. Due to the question phrasing and timing of the check-ins, people who expect to primarily sleep during the day during the 2-week EMA data collection will have their EMA data excluded from analysis as it may confound the results. Data analysis will be performed in R-studio (Posit Software, PBC, Boston, MA).

##### Reach, acceptability, usability, fidelity and sustainability

Quantitative data will be analysed using descriptive statistics. This includes both survey and website analytics data, including the following: study registration rates; proportion of registrants meeting the eligibility criteria; method of referral into the study; demographic and clinical characteristics; study attrition; number of, and which modules, were accessed; study-specific survey items about intervention acceptability and sustainability. To determine socioeconomic status and geographical location, code will be generated to match postcodes against Australian Bureau of Statistics Australian Statistical Geography Standards and Index of Relative Socio-economic Advantage and Disadvantage Socio-Economic Indexes for Areas quintiles [49].

Open-text survey responses will be collated and summarised descriptively in Microsoft Excel. Interview transcripts will be coded using NVivo and/or Microsoft Excel and analysed using thematic analysis using inductive and deductive approaches. Prior to coding the data, the researcher(s) will become familiar with the dataset by reading the transcripts and/or listening to the audio recordings.

#### Interim and additional analyses

No interim analyses are planned. No additional (e.g. subgroup) analyses are planned, but they may occur, as the data will be made available for additional research after the study findings are published (see ‘Availability of data and materials’).

### Plans to give access to the full protocol, participant level-data and statistical code

The full protocol and data management plans will be made available upon reasonable request, in writing, to the project manager or lead researcher. The participant-level quantitative data will be made available to researchers, upon reasonable request, after the study findings are published. Participants will be able to ‘opt-out’ from having their data shared/used for future unspecified research by ticking a box on the consent form. Data of participants who tick this box will not be included in the open-access meta-data or datasets shared with non-HypoPAST researchers. Statistical code may be made available upon reasonable request.

### Oversight and monitoring

#### Project coordination

The study will be coordinated by research personnel from Deakin University. The lead researcher will take overall responsibility for the study, providing guidance and oversight. A programme manager will take responsibility for day-to-day project management, overseeing that all elements of the study are implemented per-protocol and in adherence with ethics principles. For example, they will oversee data monitoring, supervise HypoPAST research personnel, conduct adverse event interviews, communicate with participants (e.g. responding to queries and complaints) and maintain communication with the studies various contributors and stakeholders (e.g. investigators and steering group, website architect, funding body). A statistician/data manager will support data monitoring (e.g. rates of eligible people recruited, randomised, withdrawn, lost to follow-up) and adverse event screening. An associate research fellow will support data entry for data monitoring, adverse event screening and coordination of the HypoPAST ‘Type 1 Diabetes Lived Experience Steering Group’ (see below). A research assistant will support communication with participants (e.g. monitoring the HypoPAST email account, responding to queries and complaints), data entry for data monitoring, maintenance of the password-protected participant codebook and other administrative duties (e.g. sending letters to health professionals, administering vouchers). Communication between the research personnel will occur weekly, via meetings and email.

#### Project oversight

Two groups oversee the HypoPAST study:

- The HypoPAST ‘Investigator Group’ comprises 14 people, including researchers and clinicians (specialising in diabetes education, endocrinology and psychology), representatives of peak bodies (for diabetes and for health professionals), and a person with lived experience of T1D. This group meets four times per year, for project oversight and to contribute their expertise into the study implementation.
- The HypoPAST ‘Type 1 Diabetes Lived Experience Steering Group’ comprises eight adults with T1D. They reviewed, and informed iterative refinements of, the intervention content and design. This involved a combination of group discussion and one-to-one cognitive debriefing interviews. The Steering Group will have continued involvement throughout the duration of the study, to contribute their expertise into the study implementation. The group meets approximately four times per year.

#### Data monitoring

Data will be monitored by the HypoPAST research personnel (see Project coordination). A data monitoring committee was not deemed necessary as the study does not involve an unapproved therapeutic good requiring a Clinical Trial Notification and trial sponsorship.

#### Adverse event reporting

The survey responses (at weeks 12 and 24), EMA responses (starting at week 24) and emails from participants to project staff will be monitored for potential adverse events. Baseline survey and EMA data will not be monitored as they are collected prior to randomisation/intervention. Serious adverse events will be defined as any severe hypoglycaemic or mental health-related event requiring medical assistance for recovery (i.e. emergency call-out, emergency department attendance and/or hospital admission). Serious adverse reactions related specifically to HypoPAST are not expected, but consideration will be given to causality. Severe hypoglycaemia or mental health-related events will only be defined as ‘serious’ if they require ambulance call-out, emergency department attendance and/or hospital admission, because the person was able to recover without medical assistance. Participants reporting any event deemed as potentially adverse will be contacted by the project manager (email and/or telephone interview) for further information to clarify the nature of the event. Adverse events will be reported to DUHREC and in the publication reporting study findings.

#### Auditing trial conduct and protocol amendments

The project is subject to independent annual financial auditing and will report annually to DUHREC regarding trial implementation. Protocol changes will be communicated to DUHREC, the funders and trial register, and reported with the study findings.

#### Funding body involvement

The Medical Research Future Fund (MRFF) Targeted Translation Research Accelerator (TTRA) did not contribute to the development of this trial protocol, and will not be involved in the conduct of the trial data collection, analysis, interpretation or write-up of findings.

#### Dissemination

Study findings will be disseminated at scientific conferences and in academic journals. A plain language summary will be published in a blog (via acbrd.org.au) and promoted via social media. Participants who opted-in to future contact will be directly emailed the plain language summary of the key findings. The primary diabetes health professional of participants who opted-in to will also be notified of the published study findings. This multi-level strategy provides several opportunities for the participants and other stakeholders (e.g. health professionals, people with diabetes) to access the findings. Participants will not be identifiable in any dissemination of the research findings. Publication authorship will be defined according to the International Committee of Medical Journal Editors criteria [50].

## Discussion

This study will provide high-quality evidence regarding the effectiveness, cost-effectiveness and acceptability of a novel, online psycho-educational programme, called HypoPAST, which is designed to reduce fear of hypoglyceamia among adults with T1D. HypoPAST draws upon considerable evidence from group-based hypoglycaemia-specific psycho-educational programmes. As it will be delivered fully online, it is expected to involve minimal cost to the health service, and enable nationally, consistent delivery and equitable access, with potential for global reach (following cultural adaptation and translations, beyond the scope of this study). As no health economic analyses have been published of group-based programmes for reducing fear of hypoglycaemia, our study will be the first to provide cost-effectiveness data, which has implications for decision-making regarding efficient resource allocation.

HypoPAST has a strong value proposition. Our work and other published research demonstrate that people with T1D (and their families) bear the daily burden of both hypoglycaemia and fear of hypoglycaemia [7, 28, 29, 51–54]. They manage self-treated and severe episodes and live with the ongoing anxiety related to the risk of such episodes, as well as the often-overlooked impacts on their productivity, sleep and quality of life [7, 27, 28, 55, 56], as well as on their family members and relationships [28, 51, 57]. We expect that the self-paced learning and experimentation approach will connect directly with participants’ real-world experiences of hypoglycaemia and fear of hypoglycaemia. Online delivery enables easy and convenient access to the programme, with no barriers in terms of time and place of delivery, which could mean broader reach for equitable and effective access and associated value, particularly for those living in rural and remote areas where internet access allows.

For health professionals, HypoPAST seeks to address a complex gap in clinical T1D care. Fear of hypoglycaemia is rarely addressed in clinical practice [27, 58], partly because hypoglycaemia has not been a major clinical focus until recent years. Importantly, a global study (24 countries, > 27,000 participants) shows that hypoglycaemia is under-reported and its impact under-estimated [59]. Face-to-face programmes are resource intensive and have not been implemented routinely, and diabetes technologies do not necessarily reduce fear of hypoglycaemia and can increase the psychological burden [15, 16, 18, 19]. Thus, health professionals do not have appropriate solutions to offer. If this programme were to be delivered face-to-face, access would likely be limited to standard ‘9-to-5’ working hours in specialist diabetes centres in metropolitan areas, and subject to waiting lists. It may also require the individual with diabetes to incur ‘out-of-pocket’ expenses to access the programme, due to the expense involved in delivering it. Our solution, if found to be effective and cost-effective, proposes maximum reach with minimal cost, as it is designed with scalability in mind. A further potential benefit of online delivery includes consistent delivery of content, reducing issues of facilitator fidelity to the curriculum, and ensuring that the programme is delivered as originally developed and intended, with minimal cost to health services.

Finally, for national diabetes organisations, HypoPAST may provide an accessible solution that they could offer direct to people with T1D to support them to live well every day with diabetes. If effective, and with appropriate implementation and roll-out, HypoPAST may enable national consistency, reach and equitable access, which is particularly important in countries such as Australia, where there is inequitable access to specialist diabetes care for people living in regional/rural communities [60].

## Trial status

This study was prospectively registered with the Australian and New Zealand Clinical Trials Registry (ACTRN12623000894695) on 21 August 2023. The study will be conducted in compliance with this protocol (Version 1.1; 24 January 2024). Participant recruitment commenced 29 January 2024 and is expected to be completed by 30 September.

## Supplementary Information

Supplementary Material 1.

Supplementary Material 2.

Supplementary Material 3.

## Data Availability

In the first instance, following database-lock, only the HypoPAST research group members responsible for data management or data analysis will have access to the de-identified data. Following publication of the findings, survey metadata will be placed in an open-access data repository (Deakin Research Data Store (RDS)), with conditional access to the de-identified dataset available to other researchers upon reasonable request. Data will be shared in a de-identified format if the Project Manager and Lead Investigator are satisfied with both the rationale for the request and how the data will be stored and used; and if researchers are willing to sign a Data Sharing Agreement documenting how they intend to use transfer, store and dispose of the data.

## Abbreviations

A2A: Attitudes to Awareness of Hypos scale
ACBRD: Australian Centre for Behavioural Research in Diabetes
AQoL-4D: Assessment of Quality of Life – 4 Dimension
CGM: Continuous glucose monitor
DUHREC: Deakin University Human Research Ethics Committee
EMA: Ecological Momentary Assessment
HFS-II: Hypoglycaemia Fear Survey II
HFS-SF: Hypoglycaemia Fear Survey II Short-Form
HIP-12: Hypoglycaemia Impact Profile
HypoA-Q: Hypoglycaemia Awareness Questionnaire
Hypo C-Q: Hypo Cues Questionnaire
HypoMETRICS: Hypoglycemia MEasurement, ThResholds and ImpaCtS
HypoPAST: Hypoglycaemia Prevention, Awareness of Symptoms, and Treatment
isCGM: Intermittent-scanned CGM, also known as ‘flash’ monitoring
mARM: Mobile Agnew Relationship Measure
NDSS: National Diabetes Services Scheme
PAID-11: Problem Areas in Diabetes scale – 11 item version
PC-PTSD-5a: The Primary Care PTSD Screen for DSM-5 – adapted for hypoglycaemia
PHQ-4: Patient Health Questionnaire
QALY: Quality-adjusted life years
T1D: Type 1 diabetes
