# Supplementary material for: Hypoglycaemia Prevention, Awareness of Symptoms, and Treatment (HypoPAST): protocol for a 24-week hybrid type 1 randomised controlled trial of a fully online psycho-educational programme for adults with type 1 diabetes
Source: Trials. 2024 Oct 29;25:725. doi: 10.1186/s13063-024-08556-1 (PMC11520494; doi:10.1186/s13063-024-08556-1)
Supplement: Supplementary file 1 — Supplementary Material 1. [file 13063_2024_8556_MOESM1_ESM.docx]

# Additional File 1: Overview of person-reported outcome measures (PROMs) and ecological momentary assessments (EMAs) used in the HypoPAST trial

| **Construct** | **Measure** | **Metric, aggregation and time point** | **Description** |  |
| --- | --- | --- | --- | --- |
| Fear of hypoglycaemia | Single item from Problem Areas of Diabetes (PAID) (1, 2) | Difference in means between groups at 24 weeks | **Eligibility screener:** The item ‘Worrying about low glucose reactions’ from the PAID (20 and 11-item versions) scale is used as part of the eligibility assessment. Response scale is the same as for the PAID-11 instrument (see below). |  |
|  | Hypoglycaemia Fear Survey II (HFS-II): Worry subscale (3) | Difference in means between groups at 24 weeks | **Primary outcome:** The 18-item Worry subscale invites respondents to indicate, on a 5-point scale ranging from 0 (Never) to 4 (Almost Always), how often they experienced concerns related to Hypoglycaemia in the past 6 months. Item scores are summed to produce a worry subscale score (range 0-72), with higher score indicating greater fear of hypoglycemia. |  |
|  | HFS-II Short-Form (HFS-SF): Avoidance and Maintain High subscales (4) | Difference in means between groups at 24 weeks | This 5-item short-form asks about behaviours undertaken to avoid hypoglycaemia or situations in which hypoglycaemia would be problematic during the past 6 months. Responses (ranging from 0 (Never) to 4 (Almost always) on a 5-point scale) are summed to form a total score, with higher scores indicating more avoidant behaviours determined by fear of hypoglycaemia. |  |
| Attitudes to awareness of hypoglycaemia; perceptions and experiences of hypoglycaemia | Attitudes to Awareness of Hypos scale (A2A; Items 4 – 19) (5) | Difference in means between groups at 24 weeks | The A2A instrument has 19 items. The first part addresses beliefs about hypoglycaemia awareness and motivation to regain awareness. The second part consist of statements reflecting beliefs about hypoglycaemia, where the participants can indicate agreement on a scale from 0 (not at all true) to 3 (very true). Three subscales can be produced (sum of relevant item scores), each representing different types of beliefs: asymptomatic hypoglycaemia normalised, hypoglycaemia concern minimised, and hyperglycaemia avoidance prioritised. Higher scores indicate greater endorsement of the belief. |  |
|  | Hypo Cues Questionnaire (Hypo C-Q; Items 2 to 40) (6) | Difference in means between groups at 24 weeks | This 40-item questionnaire assesses personal experiences and perceptions of hypoglycaemia. The questionnaire consists of 4 parts (A-D), but part A was not used in this study. Parts B to D assess the respondent’s agreement with a range of statements (e.g. related to whether the person worries more about hyperglycaemia complications than hypoglycaemia) and frequency of experiences (e.g. how frequently they are able to think clearly and act quickly when a hypo starts). Responses are ratings on a 5-point scale. Exploratory factor analysis (manuscript under development) has identified four scales: 1) low concern about hypoglycaemia (7 items), 2) hypoglycaemia burnout (6 items), 3) missing opportunities to treat hypoglycaemia (5 items), and 4) delaying treatment of hypoglycaemia (9 items). A further eight items are analysed separately. |  |
| Awareness of hypoglycaemia | Gold score (7) | Difference in means between groups at 24 weeks | Responses to this single item are rated on scale from 1 (Always aware) to 7 (Never aware). Scores ≥4 indicate impaired awareness (IAH) and scores of ≤3 indicate intact awareness. |  |
|  | Hypoglycaemia Awareness Questionnaire (HypoA-Q) Impaired Awareness subscale (Items 7-8, 10-12) (8) | Difference in means between groups at 24 weeks | Five items assess various aspects of awareness of hypoglycemia (e.g. others notice first, ‘I just know’), all rated on a 5-point scale. Scores are summed to form a total score, with higher scores indicating greater impairment of awareness. Scores ≥12 indicate impaired awareness (9). |  |
| Confidence in managing hypoglycaemia | Hypoglycaemia Confidence Scale (10) | Difference in means between groups at 24 weeks | Nine items assess the person’s confidence in their ability to manage hypoglycaemia during various situations (e.g. when exercising, when alone). The 4-point response scale ranges from 1 (not confident at all) to 4 (very confident). An average score is calculated, with scores ≥3 indicating moderate confidence. |  |
| Diabetes distress | Problem Areas Of Diabetes (PAID) 11-item scale (2) | Difference in means between groups at 24 weeks | Eleven items ask how problematic various aspects of living with diabetes are (e.g. feeling scared about living with diabetes and worrying about the future). Items are rated on a 5-point scale from 0 (not a problem) to 4 (serious problem), how problematic they consider a range of diabetes related issues. A total score is obtained by summing item scores (range 0-44) with scores ≥18 indicating severe diabetes distress. This short-form has been found to be valid and reliable among adults with type 1 diabetes. |  |
| Generalised anxiety and depressive symptoms | Patient Health Questionnaire (PHQ-4) (11) | Difference in means between groups at 24 weeks | This is a 4-item questionnaire asking about how often the respondent has experienced anxiety and depressive symptoms in the last two weeks. A 4-point response scale is used (from 0 ‘not at all’ to 3 ‘nearly every day’). The summed total score ranges from 0-12 and can be used for identifying none (0-2), mild (3-5), moderate (6-8) or severe (9-12) levels. Summed subscale score ≥3 is indicative of anxiety (item 1-2) and/or depression (item 3-4). |  |
| Hypoglycaemia frequency, severity, and awareness | HypoA-Q (items 1-4, 15 and 16) (8) | Ratio of incident rates between groups at 24-weeks for frequency of hypoglycaemia. Statistical tests (Kruskal-Wallis) comparing median number of hypoglycaemic events for each severity category between groups at 24-weeks. | Items assess frequency and severity of Hypoglycaemia, in the past week and past 6 months, while awake and while asleep. |  |
| Hypoglycaemia-specific post-traumatic stress | The Primary Care PTSD Screen for DSM-5 adapted for hypoglycaemia (PC-PTSD-5a; 5 items) (12) | Difference in means between groups at 24 weeks | This adapted version of the Primary Care PTSD for DSM-5 asks whether the person with diabetes ever had a severe hypoglycaemic episode that was unusually frightening/traumatic, and if yes, then asks 5 follow up questions about PTSD related experiences in the past month (with yes=1/no=0 response options). A total score is derived by summing the 5 items. For the original PTSD-5, a cut-off of 3 is recommended to optimise sensitivity. |  |
| Impact of hypoglycaemia on quality of life | Hypoglycaemia Impact Profile (HIP-12) (13) | Difference in means between groups at 24 weeks | Twelve items focus on the impact of hypoglycaemia on various aspect of quality of life (e.g. leisure activities, work or studies, sleep). The impact is rated from 1 (very positive) to 7 (very negative). The respondent can also indicate if an aspect of life is ‘not applicable’. A composite score is calculated by taking the average score for applicable aspects of life. Higher scores indicating greater negative impact of hypoglycaemia. The HIP-12 is an acceptable, reliable and a valid measure for assessing the impact of hypoglycaemia among adults with type 1 diabetes. |  |
| Economic evaluation | | | | |
| General health status | Assessment of Quality of Life – 4 Dimension (AQOL-4D) (14) | Difference in means (utility values and QALYs) between groups at 12 and 24 weeks | Across 12 items, this questionnaire assesses four dimensions of quality of life: independent living, relationships, mental health and senses. Respondents are asked to select the option that best describes their situation in the past week: e.g. “When doing household tasks …”, where responses range from “I need to help at all” to “I need daily help with most or all household tasks”. Utility values are calculated based on participant item scores and preference weights through available scoring algorithms. Quality adjusted life years (QALYs) are also calculated using the utility values and the area under the curve method. |  |
| Ecological momentary assessments | | | | |
| Hypoglycaemia frequency and symptoms, and daily functioning. | HypoMETRICS ’check-ins’ and ’flower motif’ (15-17) | Difference in means between groups at 24 weeks for continuous outcomes (daily functioning scores, awareness of symptoms and hypoglycaemia burden). Ratio of incident rates between groups at 24 weeks for count data (number of hypoglycaemic episodes) | A ‘check-in’ survey will be used by participants twice-daily (morning and evening) to report any episodes of hypoglycaemia they recognise via symptoms or measurement of low blood glucose (referred to as person-reported hypoglycaemia) as well as hypoglycaemia frequency, detection, response, and burden for episodes occurring during the day/while awake (6 items) and night/while asleep (7 items). The check-ins will also measure daily functioning, including fear of hypoglycaemia (2 items), fear of hyperglycaemia (2 items), sleep quality (1 item), mood (1 item) anxiety (1 item), and work and productivity (4 items). The daily functioning items has previously shown satisfactory psychometric properties in a study including people with T1D and insulin-treated type 2 diabetes (18).  A motif flower will enable participants to record all hypoglycaemia episodes. This section of the app can be completed by the participant anytime during the two-week data collection window. The flower contains 10 petals, each representing a different construct. The size of the petal, which participants change by dragging the petal in and out with one finger, represents the response options. One petal measures the timing of the hypo (response options: now, 15 minutes ago, 30 minutes ago, 1 hour ago, more than one hour ago). Another measures glucose level during the hypo (response options: Not measured, less than 2 mmol/L, 2-2.9 mmol/L, 3-3.9 mmol/L, 4mmol/L or more). The other petals each represent a different symptom of hypoglycaemia: sweating, heart palpitations, shaking, hunger, confusion, difficulty speaking, difficulty coordinating movements, and headache (response options: not at all, a little bit, somewhat, quite a bit, very much). Participants can complete the motif as many times as needed during the day or night. The motif flower has not undergone psychometric evaluation, however the symptoms used are those previously included in the validated Edinburgh Hypoglycaemia Scale (19). |  |
| Acceptability | | | | |
| Therapeutic alliance with the intervention | Mobile Agnew Relationship Measure (mARM) (20) | Descriptive statistics, measured at 12 weeks | Twenty-five items assess therapeutic alliance (i.e. the relationship between client and ‘therapist’) in digital settings. Items are rated using a 7-point response scale, ranging from 1 (strongly disagree) to 7 (strongly agree). Items 4, 8, 16, and 18 are reverse scored. |  |

Note: All measures listed are secondary outcomes unless otherwise stated.

## References

1. Polonsky WH, Anderson BJ, Lohrer PA, Welch G, Jacobson AM, Aponte JE, Schwartz CE. Assessment of diabetes-related distress. Diabetes Care. 1995;18(6):754-60.

2. Stanulewicz N, Mansell P, Cooke D, Hopkins D, Speight J, Blake H. PAID-11: A brief measure of diabetes distress validated in adults with type 1 diabetes. Diabetes Res Clin Pract. 2019;149:27-38.

3. Gonder-Frederick LA, Schmidt KM, Vajda KA, Greear ML, Singh H, Shepard JA, Cox DJ. Psychometric properties of the hypoglycemia fear survey-ii for adults with type 1 diabetes. Diabetes Care. 2011;34(4):801-6.

4. Grabman J, Vajda Bailey K, Schmidt K, Cariou B, Vaur L, Madani S, et al. An empirically derived short form of the Hypoglycaemia Fear Survey II. Diabet Med. 2017;34(4):500-4.

5. Cook AJ, DuBose SN, Foster N, Smith EL, Wu M, Margiotta G, et al. Cognitions Associated With Hypoglycemia Awareness Status and Severe Hypoglycemia Experience in Adults With Type 1 Diabetes. Diabetes Care. 2019;42(10):1854-64.

6. Little SA, Leelarathna L, Walkinshaw E, Tan HK, Chapple O, Lubina-Solomon A, et al. Recovery of Hypoglycemia Awareness in Long-standing Type 1 Diabetes: A Multicenter 2 × 2 Factorial Randomized Controlled Trial Comparing Insulin Pump With Multiple Daily Injections and Continuous With Conventional Glucose Self-monitoring (HypoCOMPaSS). Diabetes Care. 2014;37(8):2114-22.

7. Gold AE, MacLeod KM, Frier BM. Frequency of severe hypoglycemia in patients with type I diabetes with impaired awareness of hypoglycemia. Diabetes Care. 1994;17(7):697-703.

8. Speight J, Barendse S, Singh H, Little S, Inkster B, Frier B, et al. Characterizing problematic hypoglycaemia: Iterative design and preliminary psychometric validation of the Hypoglycaemia Awareness Questionnaire (HypoA-Q). Diabet Med. 2015;33(3):376-85.

9. Matus A, Flatt AJ, Peleckis AJ, Dalton-Bakes C, Riegel B, Rickels MR. Validating and Establishing a Diagnostic Threshold for the Hypoglycemia Awareness Questionnaire Impaired Awareness Subscale. Endocrine Practice.

10. Polonsky WH, Fisher L, Hessler D, Edelman SV. Investigating Hypoglycemic Confidence in Type 1 and Type 2 Diabetes. Diabetes Technol Ther. 2017;19(2):131-6.

11. Kroenke K, Spitzer RL, Williams JB, Löwe B. An ultra-brief screening scale for anxiety and depression: the PHQ-4. Psychosomatics. 2009;50(6):613-21.

12. Prins A, Bovin MJ, Smolenski DJ, Marx BP, Kimerling R, Jenkins-Guarnieri MA, et al. The Primary Care PTSD Screen for DSM-5 (PC-PTSD-5): Development and Evaluation Within a Veteran Primary Care Sample. J Gen Intern Med. 2016;31(10):1206-11.

13. Broadley M, Chatwin H, Søholm U, Amiel SA, Carlton J, De Galan BE, et al. The 12-Item Hypoglycemia Impact Profile (HIP12): psychometric validation of a brief measure of the impact of hypoglycemia on quality of life among adults with type 1 or type 2 diabetes. BMJ Open Diabetes Res Care. 2022;10(4).

14. Hawthorne G, Korn S, Richardson J. Population norms for the AQoL derived from the 2007 Australian National Survey of Mental Health and Wellbeing. Aust N Z J Public Health. 2013;37(1):7-16.

15. Søholm U, Zaremba N, Broadley M, Axelsen JL, Divilly P, Martine-Edith G, et al. Assessing the Content Validity, Acceptability, and Feasibility of the Hypo-METRICS App: Survey and Interview Study. JMIR Diabetes. 2023;8:e42100.

16. Søholm U, Broadley M, Zaremba N, Divilly P, Nefs G, Mahmoudi Z, et al. Investigating the day-to-day impact of hypoglycaemia in adults with type 1 or type 2 diabetes: design and validation protocol of the Hypo-METRICS application. BMJ Open. 2022;12(2):e051651.

17. Søholm U, Broadley M, Zaremba N, Divilly P, Baumann PM, Mahmoudi Z, et al. The impact of hypoglycaemia on daily functioning among adults with diabetes: a prospective observational study using the Hypo-METRICS app. Diabetologia. 2024. DOI: 10.1007/s00125-024-06233-1.

18. Søholm U, Broadley M, Zaremba N, Divilly P, Nefs G, Carlton J, et al. Psychometric properties of an innovative smartphone application to investigate the daily impact of hypoglycemia in people with type 1 or type 2 diabetes: The Hypo-METRICS app. PLoS One. 2023;18(3):e0283148.

19. Hepburn DA, Deary IJ, Frier BM. Classification of symptoms of hypoglycaemia in insulin-treated diabetic patients using factor analysis: relationship to hypoglycaemia unawareness. Diabet Med. 1992;9(1):70-5.

20. Berry K, Salter A, Morris R, James S, Bucci S. Assessing Therapeutic Alliance in the Context of mHealth Interventions for Mental Health Problems: Development of the Mobile Agnew Relationship Measure (mARM) Questionnaire. J Med Internet Res. 2018;20(4):e90.
