# Supplementary material for: Hypoglycaemia Prevention, Awareness of Symptoms, and Treatment (HypoPAST): protocol for a 24-week hybrid type 1 randomised controlled trial of a fully online psycho-educational programme for adults with type 1 diabetes
Source: Trials. 2024 Oct 29;25:725. doi: 10.1186/s13063-024-08556-1 (PMC11520494; doi:10.1186/s13063-024-08556-1)
Supplement: Supplementary file 2 — Supplementary Material 2. [file 13063_2024_8556_MOESM2_ESM.docx]

# Additional File 2: Semi-structured interview guide

- Interviewer introduces themselves and thanks participant
- Remind participant interview will be audio recording & take 30-45 mins
- Check whether they have any questions before starting
- Start audio recording (inform participant they are doing so).
- State date and participant ID and study name
- Commence interview

Thinking back to when you first signed-up for this study, what was it you were hoping the program would do for you? (Prompts if needed: How were you expecting the program was going to work and help you?

- To what extent did it meet your expectations? Differ from your expectations? Explore response/reasons.

Thinking about the overall HypoPAST program, what did you think about it?

- Prompts: What did you think of the content, the format, the design? how easy or difficult it was it to access and use?
- What did you like? What could be improved? Was anything missing from the program that you expected to be included?
- How did you feel about sharing your thoughts or how you manage your hypos with the program?

Let’s talk about your experiences using the HypoPAST online program. Could you tell me about how you used HypoPAST?

- Prompts: Did you use it immediately? All in one sitting? Split up over several days / weeks? When did you last use it? Did you complete the entire program or specific modules and why?

Now, let's talk about what happened after you completed the HypoPAST program

- Did you learn (or pick up) anything from HypoPAST that was useful to you in your daily life? What did you find most applicable? … most useful?
  - Prompts: Did it change anything about ...
  - ... how you **fee**l about hypos?
  - ... your **awareness** of your hypo symptoms?
  - … how you **think** about your hypo symptoms?
  - ... how you **manage** your hypos?
  - ... how **often** you have hypos?
  - ... how **severe** they are when you have them?
  - ... how **confident** you feel in managing them?

[If there has been some change noticed by the participant]

- Overall, were all the changes positive? Explain. Were there any negative experiences or changes? Were any of the changes surprising to you? Explain.
- Have they lasted? Improved over time or started to drop off? How confident are you that they will continue?
- How much effort does it take for you to maintain the changes? Has that improved or got harder over time?
- How important are these changes to you? What do they mean for you and for living with diabetes in the future?
- Have your family or friends noticed any differences in how you feel, talk about, or manage hypos ?
  - Prompt: has it had any impact on your conversations with other people about hypos? your family? your work colleagues? your health professionals? How? What is the difference?
- What do you think makes HypoPAST work for you?
- Were there any parts of the program that you expected to have an effect but they didn't? Explain
- Overall, is there anything about the program that you think could be changed that would make it work better for you?
- Is there anything you could have done differently to increase the benefits of the program for you?
- When you think back to what you were hoping to get out of the program, how well do you think it did in achieving that?

Would you recommend the program to other people?

- If this program was to be offered outside a research study, how would you like to find out about it?
- Do you think diabetes health professionals should recommend the program to people with type 1 diabetes? Why? What does it offer that you don’t get from your health professionals?

Is there anything else you'd like to tell us about the HypoPAST program?

Anything else about the study? e.g. the surveys, HypoPAST, this interview?

Thank participant for their time. Close interview.
