# Supplementary figures and images for: Hypoglycaemia Prevention, Awareness of Symptoms, and Treatment (HypoPAST): protocol for a 24-week hybrid type 1 randomised controlled trial of a fully online psycho-educational programme for adults with type 1 diabetes
Source: Trials. 2024 Oct 29;25:725. doi: 10.1186/s13063-024-08556-1 (PMC11520494; doi:10.1186/s13063-024-08556-1)

# Additional File 3: Plain language statement and consent form


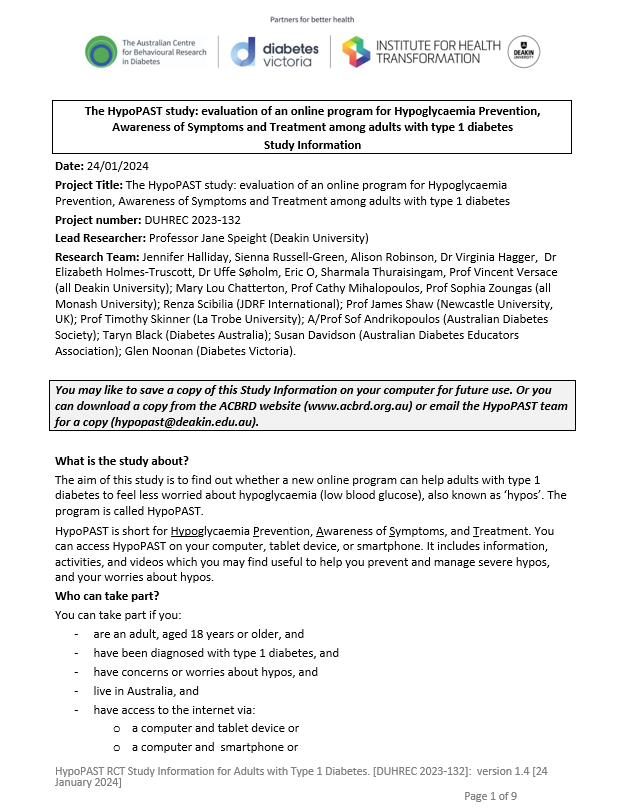


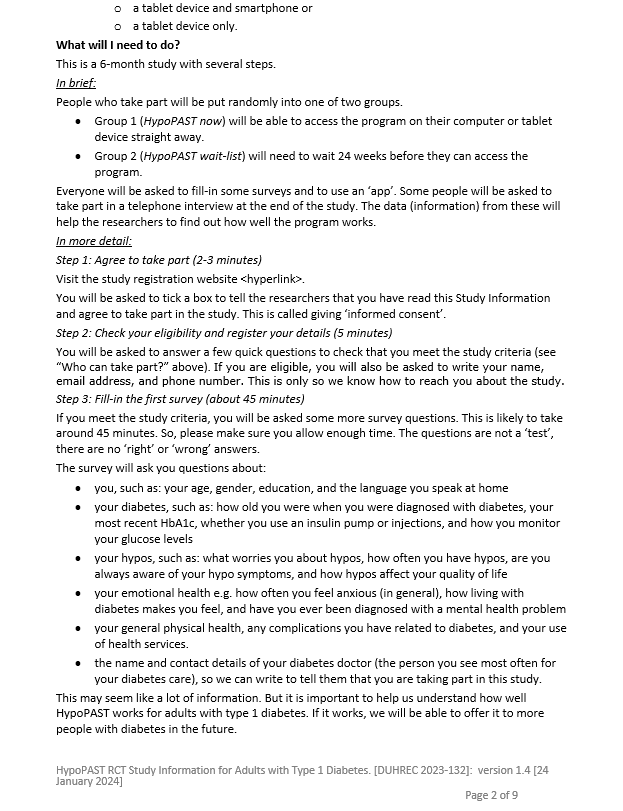


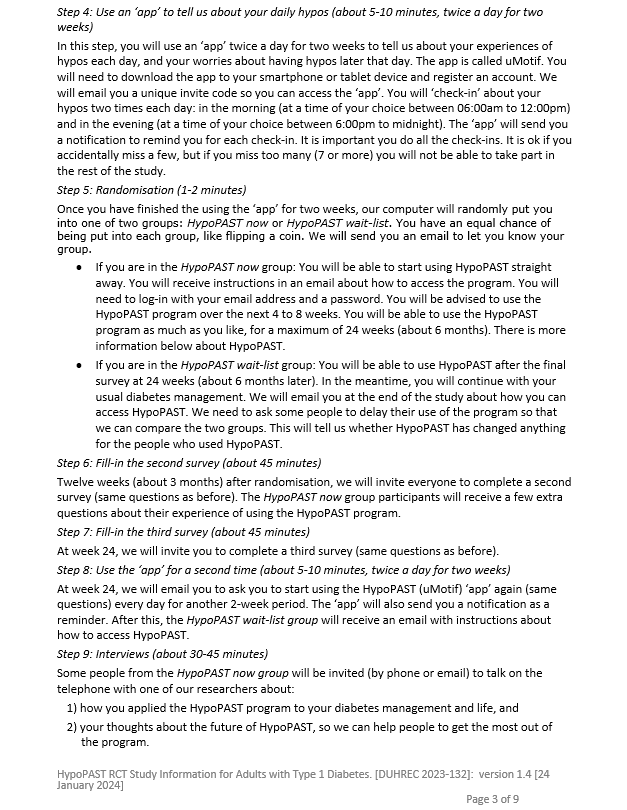


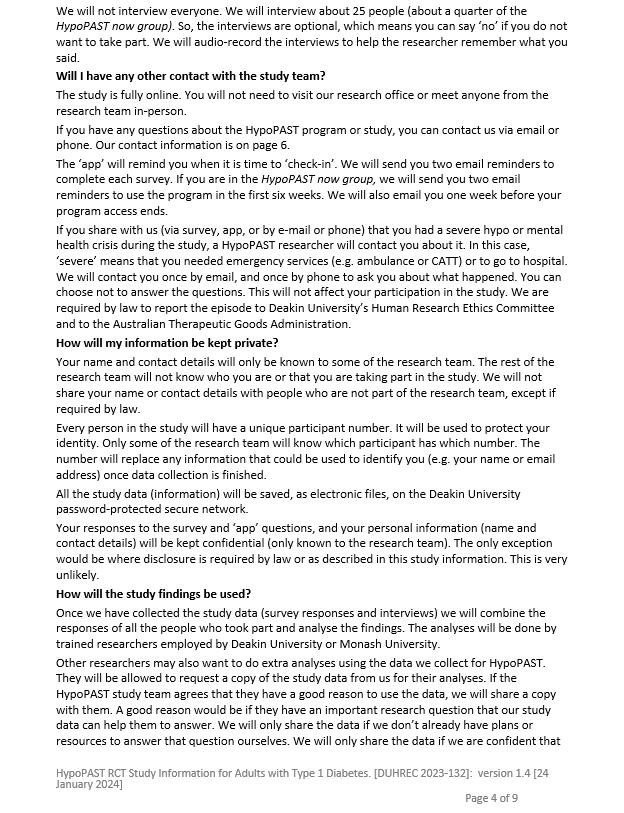


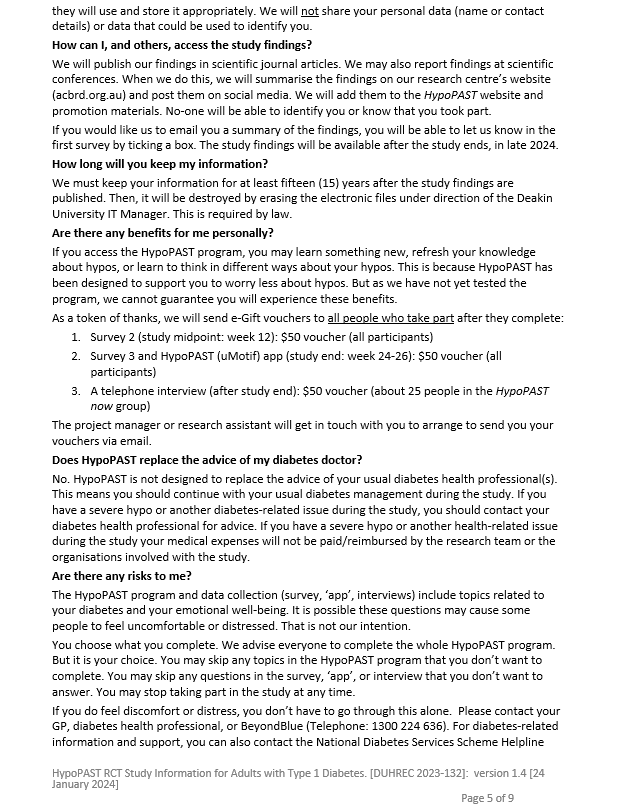


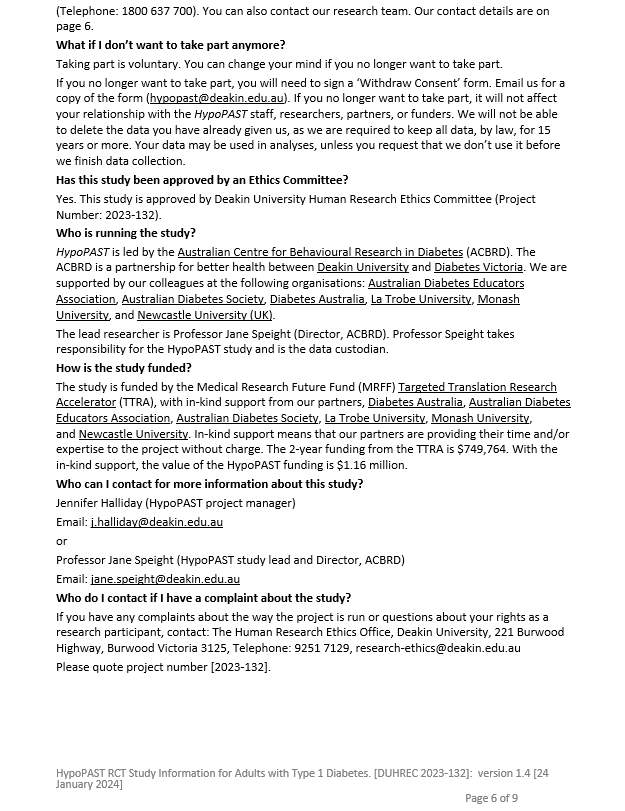


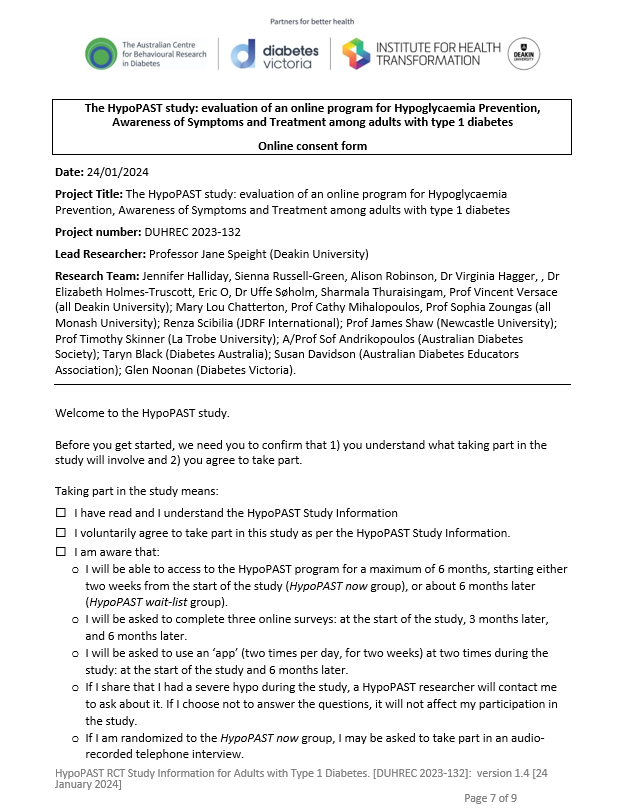


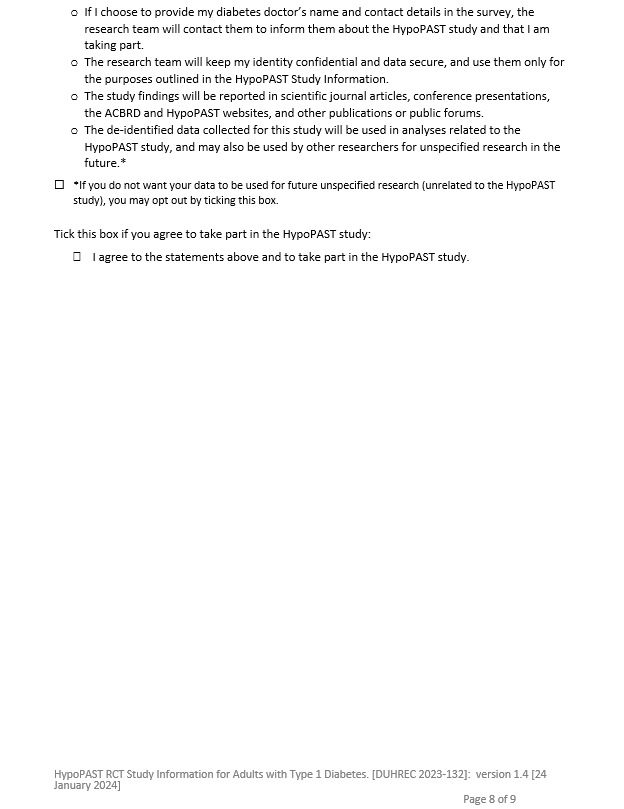


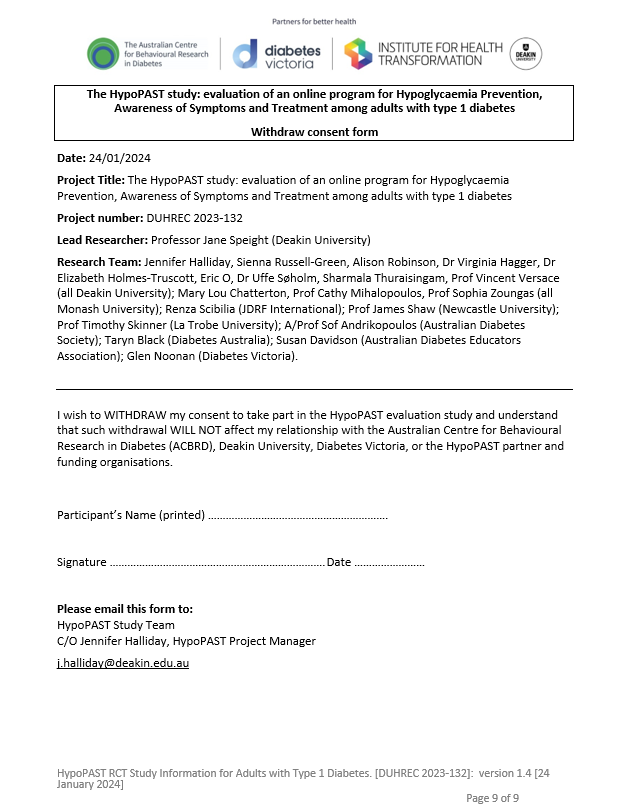

Supplement: Supplementary file 3 — Supplementary Material 3. [file 13063_2024_8556_MOESM3_ESM.docx]
